# A novel RUNX2 missense mutation predicted to disrupt DNA binding causes cleidocranial dysplasia in a large Chinese family with hyperplastic nails

**DOI:** 10.1186/1471-2350-8-82

**Published:** 2007-12-31

**Authors:** Shaohua Tang, Qiyu Xu, Xueqin Xu, Jicheng Du, Xuemei Yang, Yusheng Jiang, Xiaoqin Wang, Nancy Speck, Taosheng Huang

**Affiliations:** 1 The prenatal diagnostic Center of Wenzhou City, Department of genetics of Wenzhou No2 Hospital, Wenzhou, China; 2 Department of Biochemistry, Dartmouth Medical School, Hanover, New Hampshire, USA; 3 Department of Pediatrics, Division of Human Genetics & Metabolism, Department of Developmental and Cell Biology, Department of Pathology, University of California, Irvine, CA, USA

## Abstract

**Background:**

Cleidocranial dysplasia (CCD) is a dominantly inherited disease characterized by hypoplastic or absent clavicles, large fontanels, dental dysplasia, and delayed skeletal development. The purpose of this study is to investigate the genetic basis of Chinese family with CCD.

**Methods:**

Here, a large Chinese family with CCD and hyperplastic nails was recruited. The clinical features displayed a significant intrafamilial variation. We sequenced the coding region of the RUNX2 gene for the mutation and phenotype analysis.

**Results:**

The family carries a c.T407C (p.L136P) mutation in the DNA- and CBFβ-binding Runt domain of RUNX2. Based on the crystal structure, we predict this novel missense mutation is likely to disrupt DNA binding by RUNX2, and at least locally affect the Runt domain structure.

**Conclusion:**

A novel missense mutation was identified in a large Chinese family with CCD with hyperplastic nails. This report further extends the mutation spectrum and clinical features of CCD. The identification of this mutation will facilitate prenatal diagnosis and preimplantation genetic diagnosis.

## Background

Cleidocranial dysplasia [CCD, Min 119600] is a skeletal dysplastic disorder. Clinical features include delayed closure of skull sutures, hypoplastic or aplastic clavicles, and dental anomalies [1]. Patients often present with wide-open fontanels and middle face dysplasia, such as depressed nasal bridges. Clavicular hypoplasia can cause narrow, sloping shoulders that are often opposed at the mid line. Other clinical features include brachydactyly, tapering fingers, and short, broad thumbs and toes. Dental malformations include delayed eruption of secondary dentition and failure to shed primary teeth, which results in supernumerary teeth with dental crowding and malocclusion [2,3].

A variety of mutations in RUNX2 cause CCD [4-11], but no clear genotype-phenotype correlation has been established in CCD patients [8]. RUNX2 is an osteoblast-specific transcription factor that is a member of the core binding family (CBF) [12]. RUNX2 interacts with a non DNA-binding CBFβ subunit and the resulting complex binds to *cis*-acting elements in the promoters of genes required for skeletal formation such as osteocalcin [13]. The RUNX2-CBFβ complex serves as a master regulator of osteoblast differentiation and a scaffold that controls the assembly of transcription factors regulating skeletal gene expression [14]. In addition to osteocalcin, RUNX2 regulates vascular endothelial growth factor (VEGF) [15], COL10A1, and matrix metalloproteinase 13 (MMP13) [16]. COL10A1 is crucial for chondrocyte hypertrophy. VEGF and MMP13 are critical for hypertrophic chondrocytes, and mutations in RUNX2 result in a markedly diminished hypertrophic zone in long bone cartilage [17]. Additionally, RUNX2 and phosphatidylinositol 3-kinase (PI3K) regulate osteoblast and chondrocyte differentiation and migration in a mutually dependent manner [18]. Signal transducer and activator of transcription 1(STAT1) also interacts with RUNX2 and attenuates its transcriptional activity through cytoplasmic sequestration [19]. It has been shown that overexpression of RUNX2 results in growth arrest through p27 (KIP1)-induced inhibition of the s-phase cyclin complex, followed by dephosphorylation of the RB1 protein [20]. Since osteoblast proliferation and differentiation are inversely correlated, RUNX2 inhibits osteoblast proliferation. Here, we report a large Chinese family with CCD. We predict that a novel mutation of RUNX2 disrupts the protein-DNA interaction.

## Methods

### Patients

**Figure 1:**
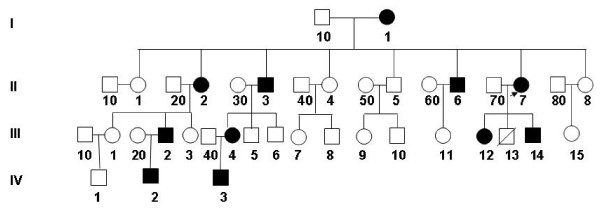
Pedigree of CCD family with hyperplastic nails. The proband (II7) is indicated by an arrow. Squares indicate males and circles indicate females. Closed symbols indicate affected individuals, and crossed lines denote deceased individuals.

**Figure 2:**
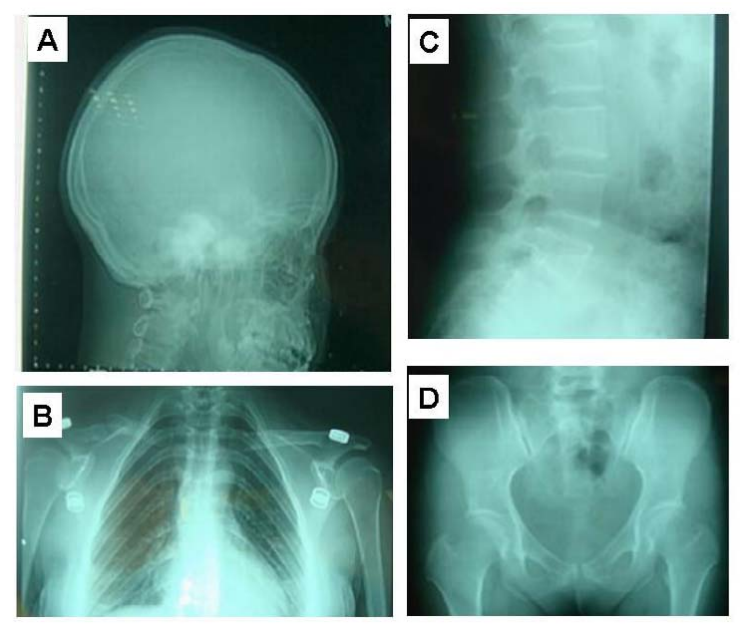
**A) **Cranial x-ray showing an asymmetrical skull, abnormal wormian bone, poor pneumatization of the sinuses, no eruption of third teeth, and a hypoplastic third tooth of the right upper jaw. **B)** Chest x-ray reveals a cone-shaped chest, high position of the scapular bone, aplasia of the lateral and middle thirds of the clavicle, and mild scoliosis between T6–T10. **C)** Vertebral x-ray showing mild dislocation of L5/S1. **D)** Pelvic x-ray showing a widening sacroiliac joint, wide pubic symphysis, hypoplastic pubic bone and normal hips.

**Figure 3:**
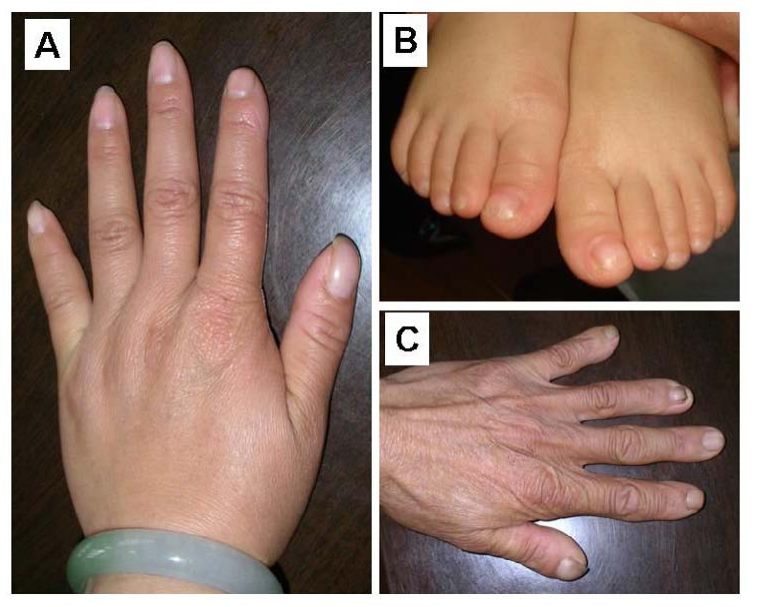
**A**, Finger nails of II-7, **B**, Toe nail, II-12 and **C**, Finger nail, I-1.

The pedigree is shown in Figure 1. The proband (II-7) is a 34-year-old G_3_P_1 _female. She received medical attention for her polyhydramnia and short stature. Upon physical examination, her height was 141.5 cm, weight 60 kg, and her arm span was 146.5 cm. She had an asymmetric face, a bossing of the forehead, hypertelorism, a depressed nasal bridge, malocclusion of teeth, a prominent lower jaw, sloping shoulders, and hyperplastic fingernails and toenails. X-ray analysis showed wormian bones and poor pneumatization of the sinuses (Figure 2A); her right cuspid tooth had not erupted and was hypoplastic. A chest x-ray revealed a cone-shaped chest, high scapular bones, aplasia of lateral and middle third of the clavicle, mild scoliosis between T6 and T10 (Figure 2B), and mild dislocation of vertebrae between L5-S1 (Figure 2C), a widening sacroiliac joint, a wide pubic symphysis, hypoplastic pubic bones (Figure 2D), normal hips. The knee and hand x-rays were normal. The family history shows that there are 11 affected individuals (6 males, 5 females) (Figure 1) from a total of 28 individuals in the family. All affected individuals have short statue, but other clinical features are very variable (Table 1). For example, several affected individuals have a normal clavicle. The cranial facial features ranged from macrocephaly to severely depressed anterior fontanels. The most striking feature of this family is that all affected individuals have hyperplastic nails (Figure 3) which is not seen in unaffected family members, and has not been previously described in CCD patients.

**Table 1:** Clinical Manifestation of Family with CCD

**Patient**	**Sex**	**Age**	**Height (cm)**	**Cranial**	**Shoulders**	**Clavicle**	**Nails**	**Other**
I1	F	76	very short	frontal bossing	sloping, bilateral		elevated nail bed	
II2	F	52	140.0	increased suture space	severe sloping, bilateral		elevated nail bed	
II3	M	49	150.5	depressed anterior fontanels	severe sloping, bilateral	absence of lateral 1/3, bilateral	elevated nail bed	
II6	M	40	152.0	frontal bossing	mild sloping, bilateral	absence of lateral 1/3, right	elevated nail bed	
II7	F	34	141.5	frontal bossing	mild sloping, bilateral	absence of lateral 1/3, right	elevated nail bed	normal ALP
III2	M	33	155.0	increased suture space	severe sloping, bilateral	absence of lateral 1/3, bilateral	elevated nail bed	
III4	F	26	134.5	severe depressed anterior fontanels	Sloping, bilateral		elevated nail bed	normal ALP
III12	F	12	120.5	frontal bossing	Sloping, bilateral	normal	elevated nail bed	
III14	M	3	80.0	frontal bossing	increased mobility, Bilateral	normal	elevated nail bed	
IV2	M	4	90.0	macrocephaly	increased mobility, Bilateral	normal	elevated nail bed	
IV3	M	2	83.5	macrocephaly	increased mobility, Bilateral	absence of lateral 1/3, right	elevated nail bed	

### Genetic analysis

Informed consent was obtained in accordance with a protocol approved by the Wenzhou 2^nd ^People Hospital Human Subject Review Board. 3 ml of blood was drawn and stored in EDTA anticoagulation tubes. Genomic DNA was purified with a genomic DNA purification kit (version 3.0, Baosheng Company, Daling, China). Genomic DNA was PCR-amplified as previously described. Eight sets of exon-specific primers were used to amplify the RUNX2 coding region [6]. The primers correspond to intron sequences, typically 30 to 50 bp away from the exon-intron boundary.

PCR was carried out using a PCR kit, following the manufacturer's instructions (Baosheng Company, Daling, China). The genomic DNA was first denatured at 94°C for 3 minutes, followed by 32 cycles of 94°C for 20 seconds, 50°C to 60°C for 30 seconds, and 72°C for 30 seconds. The PCR products were extended at 72°C for 5 minutes. The products were gel-purified with an agrose gel DNA purification kit, version 2.0 (Baosheng Company, Daling, China), and the purified PCR products sequenced using the forward and reverse primers. The sequences were analyzed with DNAStar.

## Results

**Figure 4:**
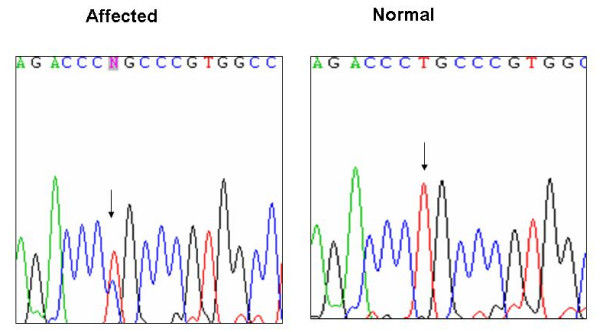
Sequencing traces showing the missense mutation in c.T407C, which causes a change from leucine to proline at amino acid 136 (p.L136P).

**Figure 5:**
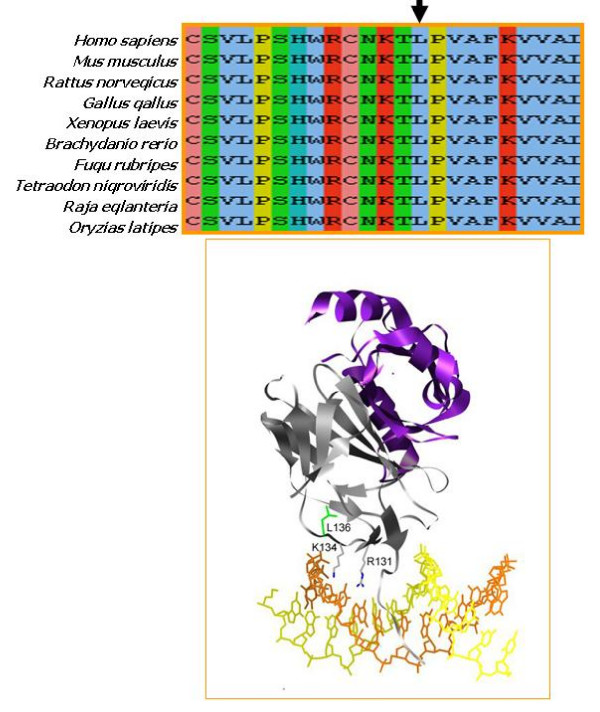
**A) **Comparison of sequences in the vicinity of RUNX2 L136 (arrow) from various species. **B**) The RUNX1 Runt domain-CBFβ-DNA complex showing the location of L136 (green). The Runt domain is grey, CBFβ is purple, and the DNA is yellow and orange. Side chains of the DNA contacting residues R131 and K134 in the βA'-B loop are also shown.

We detected a single base-pair variant in exon 1 that was verified by sequencing both strands (Figure 4). This variant in the cDNA at position 407 results in the substitution of leucine 136 with proline (Left panel). It co-segregates with affected individuals of the family. There is no such a sequence variant at that position in unaffected individuals (Right panel). More than 200 population-matched chromosomes were sequenced and no sequence variations were detected, suggesting that the sequence aberration is pathogenic. A leucine at that position is conserved in the Runt domain proteins of all vertebrates, suggesting it is functionally important (Figure 5A). The analogous residue in RUNX1 is L85, which in the crystal structures of the highly conserved RUNX1 Runt domain-CBFβ-DNA ternary complex is located in the βA'-B loop [21]. Two adjacent residues in the βA'-B loop, R131 and K134 are predicted to directly contact the DNA based on the RUNX1 structure (Figure 5B). The L136 side chain is oriented away from the DNA interface. Substitution of L136 with proline is predicted to alter the structure of the DNA-binding βA'-B loop, and thereby impair DNA binding by RUNX2. Several mutations at the DNA binding interface in RUNX1 were shown to also affect binding of CBFβ to the opposite side of the Runt domain because of their overall effect on the Runt domain structure [22] (Matheny CJ et al, unpublished data). One of these mutations was R80C, which is also located in the RUNX1 βA'-B loop (corresponding to R131 in RUNX2). Therefore, it is possible that the L136P mutation in RUNX2 would also affect CBFβ binding, but this was not directly tested.

## Discussion

Thus far, more than 70 mutations have been identified in the RUNX2 gene. The mutations are clustered in the Runt domain but occur throughout the RUNX2 protein. RUNX2 binds to DNA in a sequence-specific manner and CBFβ enhances its DNA binding affinity. Here, we identified a novel missense mutation in the Runt domain in a large Chinese family. Clinically, all affected individuals have hyperplastic nails. We show for the first time an unusual phenotype, hyperplastic nails, the molecular basis of which is unknown. This additional phenotype is most likely caused by RUNX2 mutation. However, we cannot rule out the possibility that another genetic alternation is co-segregated with the RUNX2 gene and associated with this phenotype. The most likely mechanism by which the L136P mutation affects RUNX2 function is through impaired DNA binding caused by structural alterations in the DNA-binding βA'-B loop. The identification of the L136P mutation in this family further expands the clinical phenotypes and mutation spectrum, and will contribute to prenatal molecular diagnosis and preimplantation genetic diagnoses.

Thus far, genotype-phenotype correlations have not been established for the different RUNX2 mutations found in CCD, although Zhou *et al.*[11] suggested that a T200A mutation found in a family with mild CCD might be a hypomorphic mutation. The hypomorphic nature of the T200A mutation was recently confirmed in mice [11] (Matheny CJ et al, unpublished data). There is variable expressivity of RUNX2 CCD mutations. For example, in one Italian family with a RUNX2 mutation, the reported clinical features ranged from minimal or absent clavicle to the presence of hypoplastic clavicles and delayed closure of the anterior fontanel in addition to classic craniofacial features. The family we describe here also displays significant intrafamilial phenotypic variation, indicating that there are modifying alleles at other loci. It is uncommon for CCD patients to display clinical features other than skeletal abnormalities. Two Italian CCD patients were reported to exhibit shoulder muscle abnormalities [23]. Quack et al. reported that an R225Q mutation affects nuclear RUNX2 accumulation and results exclusively in dental phenotypes. One patient with mutations resulting in an amino acid substitution at position 702 in sequences C-terminal to the Runt domain had osteoporosis, suggesting that RUNX2 not only functions in skeletal development, but also in maintaining adult bone density [6]. Extensive mutagenesis experiments have shown that many RUNX2 mutations impair DNA binding, while others affect CBFβ binding or the Runt domain structure [9,11,21,24-29] (Matheny CJ *et al.*, unpublished data).

## Conclusion

L136P is a novel mutation that most likely interferes with DNA binding by RUNX2 and would result in a nonfunctional or strongly hypomorphic allele.

## Abbreviations

CCD, Cleidocranial dysplasia; CBF, core binding family; VEGF, vascular endothelial growth factor; MMP13, matrix metalloproteinase 13; PI3K, phosphatidylinositol 3-kinase; STAT1, signal transducer and activator of transcription 1.

## Competing interests

The author(s) declare that they have no competing interests.

## Authors' contributions

ST conceived of the study, carried out the molecular genetic studies and drafted the manuscript. QX carried out the molecular genetic studies. XX participated in the sequence analysis. JD participated in the clinical data collection and X-ray analysis. XY participated in the clinical data collection. YJ participated in the sequence analysis. XW participated in design and coordination. NS participated in 3-D modeling and manuscript preparation. TH participated in the experimental design, coordinate the project and manuscript preparation.

## Pre-publication history

The pre-publication history for this paper can be accessed here:


